# The role of herbal medicines in treatment of urinary tract diseases

**Published:** 2014-01-01

**Authors:** Ahmad-Reza Gohari, Soodabeh Saeidnia

**Affiliations:** ^1^Medicinal Plants Research Center, Faculty of Pharmacy, Tehran University of Medical Sciences, Tehran, Iran; ^2^Division of Pharmacy, College of Pharmacy and Nutrition, University of Saskatchewan, Saskatoon, Canada

**Keywords:** Kidney diseases, Herbal medicines, Benign prostatic hyperplasia

**Implication for health policy/practice/research/medical education:**

Herbal medicines or remedies are applied to treat renal disorders and most of them have been proved to be useful. The medical management of nephrolithiasis is either costly or not without side effects. However, there is a common belief that using medicinal plants is safer than synthetic drugs, even though medicinal plants and other natural compounds may also result in adverse effects and the safety of these remedies under question.

Urinary diseases have affected humankind since ancient time and can persist, with serious medical consequences, throughout the world. Additionally, the incidence of renal disorders especially kidney stones have been enhanced in western countries in the last decades, in relation with economic development. It seems that acute and chronic renal failure and lithiasis (stone formation) occurred due to both nephrolithiasis (stone formation in kidney) and urolithiasis (stone formation in ureter or bladder even both places). Nowadays, alongside synthetic drugs, herbal medicines play a prominent role in treatment and prevention of various diseases. A number of herbal medicines and remedies have been reported for its significant nephroprotective activity, which is probably due to the presence of flavonoids and other effective secondary metabolites in those medicinal plants. Furthermore, literature reveals that the extracts from the leaves and aerial parts of some plants exhibit good potentials to improve kidney damages. Actually among different kidney disorders, there are two main urologic indications for application of herbal drugs as follows (1-3):

1. Inflammatory urinary tract diseases including renal gravel and more severe types of lithiasis, in which herbal remedies mainly of kidney & bladder teas have been used. As a matter of fact, urinary tract inflammation can be treated mainly by medicinal teas that possess a diuretic activity because of the consumption of the water that is ingested by drinking the teas. Actually, in patients with urinary tract infections or stone-related (and other) inflammatory irritations of the urinary tract, increasing the output of a hypo-osmolar urine is observed to be an effective way to clear ascending bacteria, crystallization nuclei, and other inflammatory agents (2).
2. Benign Prostatic Hyperplasia (BPH), for which there are three pathogenic hypothesis as below:

A. Increase in the prostatic synthesis of dihydrotestosterone (DHT) & estrogen:androgen ratio
B. Elevation in the binding capacity of sex hormone-binding globulin (SHBG)
C. Elevation in the levels of inflammatory mediators (prostaglandins & leukotrienes).

However, in BPH, saw palmetto berries, nettle root and pumpkin seeds are widely employed (2).

Among the various medicinal plants, *Arctostaphylos uva-ursi* (Ericaceae) with chemical composition of arbutin (5-12%), hydroquinone and tannins (10-20%) is well known for treatment of bacterial inflammatory diseases with three gram of the dried herb, or 400-800 mg of hydroquinone derivatives, four times daily (2,4). Moreover, *Ammi visnaga* (Apiaceae) has been traditionally used to relieve the pain of renal colic and spastic urinary tract disorders (5,6). Another medicinal herb in this regard, *Serena repens* (Palmae) is a native plant in USA and consists of lipophilic composition like saturated and unsaturated fatty acids, which has been administered in micturition difficulties associated with stage I-II in BPH with 1-2 g of the crude drug or 320 g dried herb, as daily dosage (2).

*Urtica dioica* (Urticaceae) is another famous herbal drug, with composition of phytosterols, triterpenic acids, lignans, polysaccharides and simple phenol compounds, employed in micturition difficulties associated with stage I-II of prostatic adenoma. Its mechanism of action is defined by interacting (competitive displacement) with SHBG and multiple inhibitory effects on inflammatory mediators (acid polysaccharide fraction). The daily dosage of this plant is 4-6 g of crude drug, although some adverse effects such as gastrointestinal disturbances have been reported (2,7). Furthermore, *Cucurbita pepo* (Cucurbitaceae) with chemical composition of fatty acids (linoleic acid, 64%), phytosterols, tocopherols and carotenoid is a useful plant in urinary tract diseases. Micturition difficulties associated with stage I-II of prostatic adenoma can be one of its indications with daily dose of 10 g of ground seeds (2-7). Besides these famous plants, grass pollens (from *Secale cereale, Phleum pretense* and *Zea mays*), phytosterols (from *Hypoxis rooperi*), and pygeum (*Prunus africana*) are also mentioned in literature for their effectiveness in renal diseases. Some other plants as corrective herbs are used in kidney teas formulations including fennel seed, licorice root, calendula flowers and peppermint leaves (2,4).

As a matter of fact, the compound beta-sitosterol, which is widespread in many plants as well as fungi and marine organisms such as brown and red algae, has been well documented for its effectiveness in treating both BPH and prostatic cancer. Beta-sitosterol is not only the main active component of many renal-effective herbal medicines such as saw palmetto and pygeum, but also was used as a pure compound in many clinical trials and its efficacy and safety has been proved (8).

## Conclusion

Taking together, many herbal medicines or remedies are applied to treat renal disorders and most of them have been proved to be useful. The medical management of nephrolithiasis is either costly or not without side effects. However, there is a common belief that using medicinal plants is safer than synthetic drugs, even though medicinal plants and other natural compounds may also result in adverse effects and the safety of these remedies under question.

## Authors’ contributions

ARG wrote the draft. SS prepared the final manuscript.

## Conflict of interests

The authors declared no competing interests.

## Ethical considerations

Ethical issues (including plagiarism, misconduct, data fabrication, falsification, double publication or submission, redundancy) have been completely observed by the authors.

## Funding/Support

None.
